# A Novel Nucleic Lateral Flow Assay for Screening *phaR*-Containing *Bacillus* spp.

**DOI:** 10.4014/jmb.1907.07045

**Published:** 2019-10-25

**Authors:** Nay Yee Wint, Khine Kyi Han, Wariya Yamprayoonswat, Pattarawan Ruangsuj, Supachoke Mangmool, Chamras Promptmas, Montri Yasawong

**Affiliations:** 1 Department of Biochemistry, Faculty of Pharmacy, Mahidol University, Bangkok 0400, Thailand; 2 Department of Pharmacology, Faculty of Pharmacy, Mahidol University, Bangkok 10400, Thailand; 3 Chulabhorn Graduate Institute, Chulabhorn Royal Academy, Bangkok 10210, Thailand; 4 Department of Biomedical Engineering, Faculty of Engineering, Mahidol University, Nakhon Phathom 73170, Thailand; 5 Center of Excellence on Environmental Health and Toxicology (EHT), Office of Higher Education, Bangkok 10400, Thailand

**Keywords:** DNA lateral flow, PHA synthase, *phaR*, PHA, *Bacillus*

## Abstract

Polyhydroxyalkanoate (PHA) synthase is a key enzyme for PHA production in microorganisms. The class IV PHA synthase is composed of two subunits: PhaC and PhaR. The PhaR subunit, which encodes the *phaR* gene, is only present in class IV PHA synthases. Therefore, the *phaR* gene is used as a biomarker for bacteria that contain a class IV PHA synthase, such as some *Bacillus* spp. The *phaR* gene was developed to screen *phaR*-containing *Bacillus* spp. The *phaR* screening method involved two steps: *phaR* gene amplification by PCR and *phaR* amplicon detection using a DNA lateral flow assay. The screening method has a high specificity for *phaR*-containing *Bacillus* spp. The lowest amount of genomic DNA of *B. thuringiensis* ATCC 10792 that the *phaR* screening method could detect was 10 pg. This novel screening method improves the specificity and sensitivity of *phaR* gene screening and reduces the time and cost of the screening process, which could enhance the opportunity to discover good candidate PHA producers. Nevertheless, the screening method can certainly be used as a tool to screen *phaR*-containing *Bacillus* spp. from environmental samples.

## Introduction

Plastics play a critical role in daily life because of their attractive properties and wide range of applications [1]. The increased rate of plastic consumption has led to environmental problems because plastic waste is difficult to manage as its degradation rate is very low in the environment. The cost of plastic waste management has increased because of the requirement for landfill space and energy for recycling processes. Broken-down plastic waste produces microplastics that are known to harm wild animals, especially marine organisms, which mistake them for food. Microplastics can also be consumed by humans via drinking water and food. Although the risk to humans is still unclear, the smallest microplastics can enter the bloodstream [2]. Thus, biodegradable plastics have been developed to overcome environmental issues caused by traditional plastics. Biodegradable plastics can be mineralized by microorganisms in the environment [3]. Some bioplastics can be produced from bacteria and archaea, such as polyhydroxyalkanoate (PHA), polylactic acid (PLA) and polybutylene succinate (PBS) [4, 5]. Among bioplastics, PHAs are easily degraded to carbon dioxide and water, or to water and methane depending on the concentration of oxygen [4]. PHAs are a promising biomaterial for the development of environmentally friendly biodegradable plastics. PHAs can be synthesized by many bacteria and some archaea under imbalanced growth conditions such as excess carbon and limited availability of essential nutrients including nitrogen, phosphorus, and oxygen for growth [5-6]. The key enzymes in PHA polymerization are PHA synthases, which can be classified into four main classes depending on their structures, substrate specificities and subunit components [6]. The class IV PHA synthase is encoded by the *phaC* and *phaR* genes and catalyzes the polymerization of short-chain-length monomers (C_3_ - C_5_) [7]. Class IV PHA synthases can be found mostly in bacteria that belong to the genus *Bacillus* [8]. The genus *Bacillus* is one of the largest bacterial groups of the family *Bacillaceae*. The *Bacillus* genus contains 379 species and 7 subspecies [9-10]. *Bacillus* are found in various types of environments, such as air, soil and water. Most of the bacterial species in the *Bacillus* genus are nonpathogenic bacteria [11]. *Bacillus* spp. are reported to be the most versatile PHA producers [12, 13]. They can use a wide variety of carbon sources for PHA production, including corn steep liquor [14], crude glycerol [15], molasses [14] and wastewater from the sugar industry [8]. Some bacterial strains of the *Bacillus* genus, such as *B. cereus* [16], *B. megaterium* [14], *B. mycoides* [17], *B. subtilis* [8] and *B. thuringiensis* [15], have been reported as candidate PHA producers. PHA-producing *Bacillus* are of interest due to their ability to use a wide variety of substrates for growth and to produce PHAs. This will be a very economical method for bioplastic production [15].

There are a variety of methods available to identify PHA producers. Functional screening methods such as Sudan Black staining [18], Nile Blue A staining and direct staining of bacterial colonies by fluorescence dye [19] are useful methods for the detection of PHA granules that have been accumulated in cells. However, these functional screening methods require time, specific media and growth conditions and the screening of candidate microorganisms for PHA production. False negative results were observed during screening when the candidate microorganisms were grown in nonoptimized media or conditions [20].

Thus, polymerase chain reaction (PCR) can be used as a genetic screening method for the detection of important gene markers of PHA-production pathways [21, 22]. The PCR method shows high specificity to the target biomarker genes and allows amplification of the target genes at low concentrations [21, 22]. However, postanalysis including gel electrophoresis is required after PCR. This step is time consuming and requires specific equipment. Thus, a simple and fast method for amplicon detection, such as a DNA lateral flow assay, can be applied instead of gel electrophoresis.

As mentioned above, the combination of PCR and a DNA lateral flow assay was developed as a method for the detection of the *phaR* gene of PHA-producing *Bacillus* spp.

## Materials and Methods

### Bacterial Strains and Culture Method

*Bacillus cereus* ATCC 14579 and *Bacillus thuringiensis* ATCC 10792 were purchased from American Type Culture Collection (ATCC). *Cupriavidus necator* DSM 428, *Pseudomonas aeruginosa* DSM 19880, genomic DNA of *Allochromatium vinosum* DSM 180 and genomic DNA of *Haloquadratum walsbyi* DSM 16854 were obtained from the German Collection of Microorganisms and Cell Cultures (DSMZ). *Escherichia coli* ATCC 25922 and *Staphylococcus aureus* ATCC 25923 were obtained from the Department of Microbiology, Faculty of Pharmacy, Mahidol University. Bacteria were inoculated in LB broth and incubated at a temperature between 30-37°C with shaking at 250 rpm overnight.

### Primer Design for the *phaR* Gene of the *Bacillus* spp.

The *phaR* gene sequences of bacteria were obtained from the GenBank genome database. A multiple sequence alignment was performed with complete *phaR* gene sequences based on a progressive alignment method using Clustal X version 1.81 [23]. The aligned sequences of the *phaR* gene were used as input data for phylogenetic tree analysis. The conserved regions of the *phaR* gene were selected for the primers designed. A neighbor-joining (NJ) tree of the *phaR* gene was constructed using MEGA version 6 [24]. The *phaR* phylogenetic tree was visualized by TreeView version 1.6 [25].

### PCR Analysis

Genomic DNA (gDNA) was extracted using a DNeasy Blood and Tissue Kit (Qiagen, Germany). The concentration of gDNA was determined with a Nanodrop spectrophotometer (Thermo, USA). *B. cereus* ATCC 14579 and *B. thuringiensis* ATCC 10792 were used as positive control samples because each strain contained the *phaR* gene. The *phaR* primers were purchased from Bio Basic Canada Inc. The forward primer (BGPhaR-F) contained biotin at the 5' end and the reverse primer (BGPhaR-R) contained FITC at the 5' (Table 1). The size of the PCR product was approximately 122 bp. The PCR was performed in a total volume of 50 μl consisting of 40.2 μl of deionized water, 5 μl of 10×Vi Buffer S (Vivantis, Malaysia), 2 μl of 80 μM dNTPs (Vivantis), 1 μl of 10 μM BGPhaR-F (Bio Basic), 0.5 μl of 10 μM BGPhaR-R (Bio Basic), 1 μl of 10 ng/μl DNA template and 0.3 μl of 5 U/μl *Taq* DNA polymerase (Vivantis). The PCR conditions started with an initial step at 94°C for 2 min, followed by 35 cycles of 94°C for 30 s, 54°C for 30 s, and 72°C for 30 s, and a final step of 72°C for 1 min. PCR was performed on a T100 Thermal Cycler (Bio-Rad, USA). The reproducibility of the PCRs was examined by testing three replicates of genomic DNA by each assay and repeating the experiment five times. The amplicon was analyzed by gel electrophoresis in 1% w/v of agarose gel at 80 V for 35 min. GeneRuler 100 bp Plus DNA Ladder (Thermo) was used as the DNA marker for agarose gel electrophoresis. PCR products were visualized with a UV transilluminator (Syngene, England).

### Specificity Test of the *phaR* Screening Method

The details of the microorganisms used in this study are shown in Table 2. *B. cereus* ATCC 14579 and *B. thuringiensis* ATCC 10792 were used as positive controls because they are able to produce PHAs and contain the *phaR* gene. A no-template control (NTC) reaction was performed using deionized water instead of the DNA template. *E. coli* ATCC 25922 and *S. aureus* ATCC 25923 were used as negative controls for group 1 (NC1) because they cannot produce PHAs and lack the *phaR* gene. Negative control group 2 (NC2) included *A. vinosum* DSM 180, *C. necator* DSM 428, *H. walsbyi* DSM 16854 and *P. aeruginosa* DSM 19880, which are all capable of producing PHAs but lack the *phaR* gene. The *phaR* screening method consisted of two steps, amplification and detection. First, the *phaR* gene of *Bacillus* spp. was amplified by PCR. Then, the *phaR* amplicon was observed on the HybriDetect DNA lateral flow assay (Milenia Biotec, Germany). The specificity of the *phaR* screening method was depended on the amplification step. PCR amplification was positive when the amplicon contained biotin and FITC at its flanking region. The DNA lateral flow assay was set up according to the instructions provided with the kit. One microliter of PCR product was added to the DNA lateral flow strip. The results appeared on the DNA lateral flow strip within one minute. One line on the strip (control line) indicated that no amplification of the *phaR* gene was detected. The *phaR* amplicon was detected when two lines appeared on the strip (control line and test line).

### Sensitivity Test of the *phaR* Screening Method

Genomic DNA of the positive control samples was diluted to 1,000 pg/μl, 100 pg/μl and 10 pg/μl. Each dilution of the gDNA was used as a DNA template for the PCR as previously described. The PCR products were examined with 1% w/v agarose gel electrophoresis and the DNA lateral flow assay (Milenia Biotec).

### Screening *phaR* Gene of *Bacillus* spp. from Environmental Samples

Soil samples were collected from a municipal landfill of Yangon, Myanmar (16°52'22.1"N 96°11'56.0"E). The soil samples were kept in 50 ml sterile tubes and stored at 4°C. One gram of soil was resuspended in 10 ml of LB broth. Then, 100 μl of the suspension was spread onto LB agar (Titan, India) and cultured at 30°C overnight. Different single colonies were selected based on their morphological characteristics. The selected colonies were grown in LB broth at 30°C with shaking at 250 rpm overnight. The gDNA of the isolated strains was obtained using a DNeasy Blood and Tissue Kit (Qiagen). Then, the *phaR* genes were amplified by the PCR method, and the amplicon was detected on the HybriDetect DNA lateral flow strip (Milenia Biotec).

Sequencing of the *phaR* amplicon was performed using the dideoxy method (1^st^ Base, Singapore). The partial sequences of the *phaR* gene were subjected to multiple sequence alignment based on the progressive alignment method using Clustal X version 1.81 [23]. The NJ tree of the *phaR* gene was constructed based on the maximum composite likelihood model using MEGA version 6 [24].

## Results and Discussion

### Primer Design for the *phaR* Gene of the *Bacillus* spp.

The details of the *phaR* gene that were selected for this study are shown in Table S1. The length of the *phaR* genes of the *Bacillus* spp. were 483 to 600 bp. The NJ tree of the *phaR* gene (Fig. S1) was constructed based on the maximum composite likelihood model. The conserved region of *phaR* genes was used to design primers. Based on the consensus sequence of the alignment, the conserved region of position 70-91 was selected for the design of the forward primer, and the conserved region of position 169-192 was selected for the design of the reverse primer (Fig. S2). The details of the *phaR* primers are shown in Table 1.

### PCR Analysis

PCR was performed for validation of the *phaR* primers (Table 1). The PCR was optimized to achieve the highest specificity of the primers and the shortest analysis. The PCR successfully amplified 122 bp of the *phaR* gene of *B. cereus* ATCC 14579 and *B. thuringiensis* ATCC 10792, and less than 100 min was required for the PCR analysis. Nonspecific amplification and primer dimers were not observed.

### Specificity Test of the *phaR* Screening Method

**Fig. 1:**
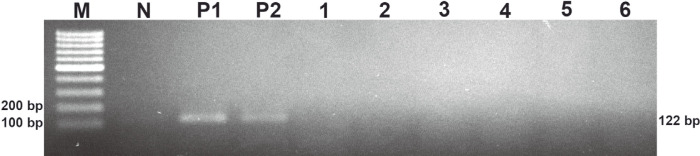
Analysis of the specificity of the *phaR* PCR. (M) DNA marker, (N) no-template control, (P1) positive control (*B. cereus* ATCC 14579), (P2) positive control (*B. thuringiensis* ATCC 10792), (1) *E. coli* ATCC 25922, (2) *S. aureus* ATCC 13565, (3) *A. vinosum* DSM 180, (4) *C. necator* DSM 48, (5) *H. walsbyi* DSM 16854, and (6) *P. aeruginosa* DSM 19880.

**Fig. 2:**
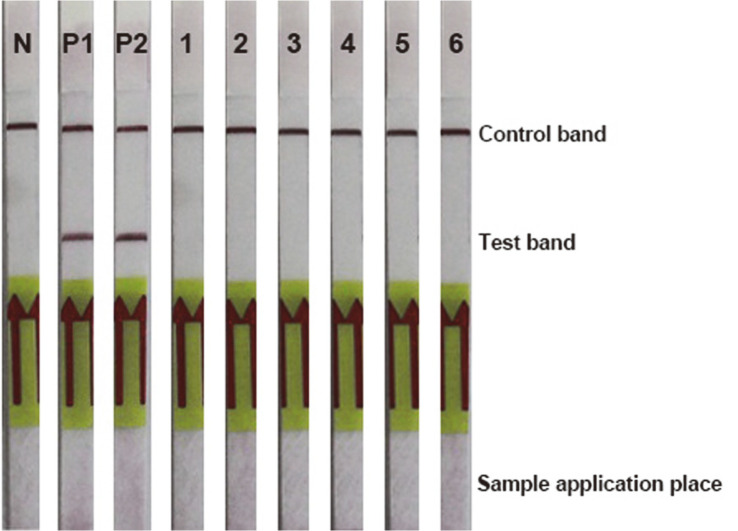
Detection of the *phaR* amplicon with the DNA lateral flow assay. (N) No-template control, (P1) positive control (*B. cereus* ATCC 14579), (P2) positive control (*B. thuringiensis* ATCC 10792), (1) *E. coli* ATCC 25922, (2) *S. aureus* ATCC 13565, (3) *A. vinosum* DSM 180, (4) *C. necator* DSM 48, (5) *H. walsbyi* DSM 16854, and (6) *P. aeruginosa* DSM 19880.

The PCR products of the *phaR* amplification are shown in Fig. 1. There was no PCR product for NTC, NC1 and NC2. No nonspecific amplifications or primer dimers were observed in any PCR amplifications. The PCR products were tested with a DNA lateral flow assay, and these results are shown in Fig. 2. The control line and test line were observed with DNA lateral flow when tested with the amplicons of the positive controls (*B. cereus* ATCC 14579 and *B. thuringiensis* ATCC 10792). However, only the control line was observed with DNA lateral flow when tested with the amplicons of NC1 and NC2.

### Sensitivity Test of the *phaR* Screening Method

**Fig. 3:**
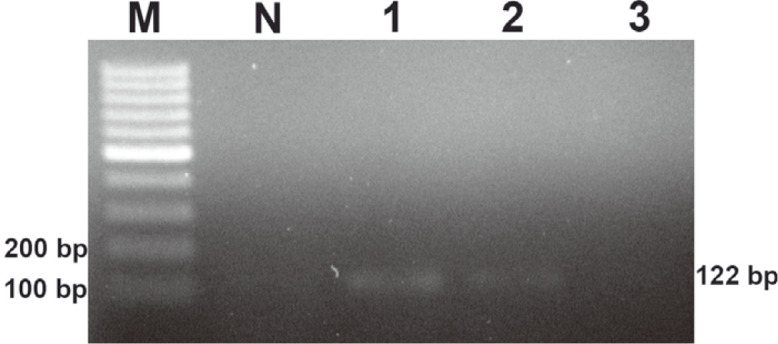
Analysis of the sensitivity of the *phaR* PCR for *B. cereus* ATCC 14579; (M) DNA marker, (N) notemplate control, (1) 1,000 pg, (2) 100 pg and (3) 10 pg.

**Fig. 4:**
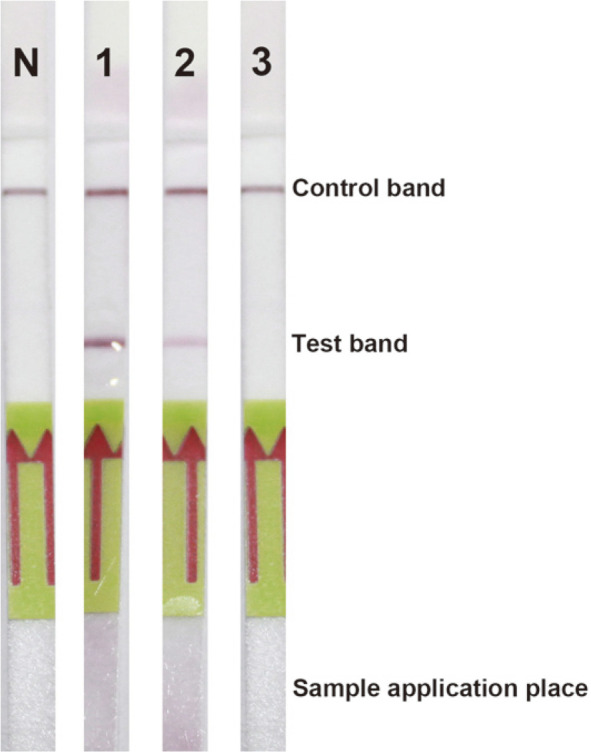
Detection of the *phaR* amplicon of *B. cereus* ATCC 14579 with the DNA lateral flow assay; (N) notemplate control, (1) 1,000 pg, (2) 100 pg and (3) 10 pg.

**Fig. 5:**
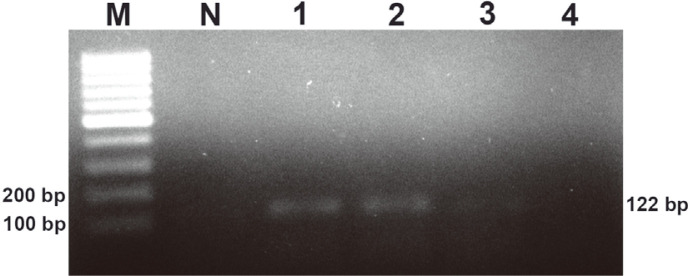
Analysis of the sensitivity of the *phaR* PCR for *B. thuringiensis* ATCC 10792, (M) DNA marker, (N) notemplate control, (1) 1,000 pg, (2) 100 pg, (3) 10 pg and (4) 1 pg.

**Fig. 6:**
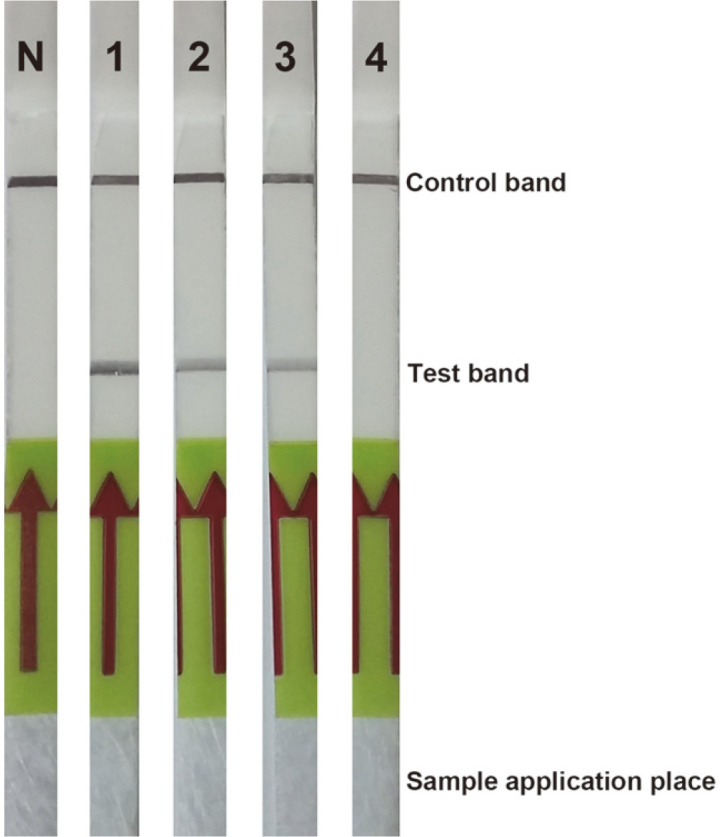
Detection of the *phaR* amplicon of *B. thuringiensis* ATCC 10792 with the DNA lateral flow assay; (N) no-template control, (1) 1,000 pg, (2) 100 pg, (3) 10 pg and (4) 1 pg.

The *phaR* amplicon of *B. cereus* ATCC 14579 is shown in Fig. 3 with the agarose gel electrophoresis and Fig. 4 with the DNA lateral flow assay. The lowest concentration of the DNA template that the PCR and DNA lateral flow assay could detect was 100 pg. The *phaR* amplicon of *B. thuringiensis* ATCC 10792 is shown in Fig. 5 with the agarose gel electrophoresis and Fig. 6 with the DNA lateral flow assay. The lowest concentration of the DNA template that the PCR and DNA lateral flow assay could detect was 10 pg.

### Screening *phaR* Gene of *Bacillus* spp. from Environmental Samples

**Fig. 7:**
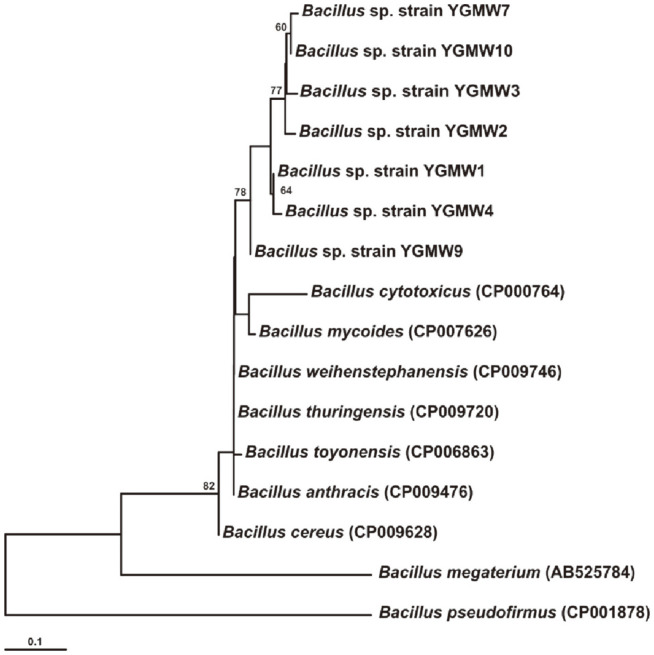
Neighbor-joining tree of the *phaR* gene of *Bacillus* spp. that were isolated from the soil of a municipal landfill of Yangon, Myanmar. The values associated with nodes correspond to the bootstrap value in %.

Sixteen isolated strains were obtained from the soil of a municipal landfill of Yangon, Myanmar. However, seven bacterial strains contained the *phaR* gene (Table 3) after screening with the *phaR* PCR combined with the DNA lateral flow assay. The *phaR* amplicon of the positive clones was sequenced, and phylogenetic analysis was performed. The *phaR* genes of the isolated strains were affiliated with bacterial strains in the genus *Bacillus* (Fig. 7).

## Discussion

Bacillus spp. are very attractive candidates for PHA production. However, more than three hundred species of the *Bacillus* genus still have not been investigated for PHA production. Screening PHA-producing *Bacillus* spp. using classical microbiological methods is a time-consuming process that requires appropriate media and optimal growth conditions for inducing PHA accumulation in cells [26]. Thus, Shamala and colleagues [26] developed a semi-nested PCR method for detection of the *phaC* gene, which encodes the PhaC subunit of the class IV PHA synthase [6]. The primers for the semi-nested PCR were designed from a single nucleotide sequence of the *phaC* gene of *B. megaterium*. This method successfully amplified the *phaC* gene of the ten standard *Bacillus* strains [26]. The class IV PHA synthase is a heterodimer that contains PhaC and PhaR subunits [6]. The PhaR subunit is presented only in class IV PHA synthase. It is encoded by the *phaR* gene, which can be used as a biomarker for class IV PHA synthases. In the current study, sixty-four *phaR* gene sequences from *Bacillus* strains were used to design primers. This ensured that the primers covered the conserved regions of the *phaR* gene of *Bacillus* spp. This may have increased the possibility of successful rates of *phaR* screening from PHA-producing *Bacillus* spp. Moreover, the *phaR* PCR required less time than the *phaC* semi-nested PCR that was described by Shamala and colleagues [26]. Because there is only one step for amplification of the *phaR* gene, the amplicon can be immediately detected with the DNA lateral flow assay. Therefore, the *phaR* screening method comprises two major steps: *phaR* gene amplification by PCR and *phaR* amplicon detection with a DNA lateral flow assay. The *phaR* screening results demonstrated highly specific detection of the *phaR* gene in *B. cereus* ATCC 14579 and *B. thuringiensis* ATCC 10792 (Figs. 1-2). Based on the results of the sensitivity test of the *phaR* screening method, the lowest amount of gDNA (*B. thuringiensis* ATCC 10792) that the screening method could detect was 10 pg (Figs. 5-6). The *phaR* screening method can be applied to screen *Bacillus* spp. isolated from soil samples (Table 3). The similarity of the *phaR* genes of the isolated *Bacillus* strains was 94-96%, and the closest relative was *B. thuringiensis* (Table 3). Kumar and colleagues have reported that *B. thuringiensis* EGU45 can utilize high concentrations of crude glycerol for PHA co-polymer production and can produce PHAs in the medium, which contains high nitrogen concentrations [15].

The *phaR* screening method is a novel assay for screening phaR-containing *Bacillus* spp. The chance of discovering a novel *phaR* gene or PHA-producing *Bacillus* spp. may increase with the use of the *phaR* screening method. Nevertheless, the *phaR* screening method can certainly be used as a tool for screening for phaR-containing *Bacillus* spp. that have been isolated from environmental samples.

## Supplemental Material

Supplementary data for this paper are available on-line only at http://jmb.or.kr.

## Figures and Tables

**Table 1:** The primers used to screen the *phaR* gene of *Bacillus* spp.

Primer	Sequence (5’→3’)	Length (bp)	5’ Labeled
BGPhaR-F	GATCCAYTWCAAGCATGGAAA	21	Biotin
BGPhaR-R	TTCAAATCTAGAACRYTKCCCAT	23	FITC

**Table 2:** Specific details of the microorganisms used in this study.

No	Species	PHA synthase	*phaR*	Designated
1	*B. cereus* ATCC 14579	Class IV	Yes	Positive control
2	*B. thuringiensis* ATCC 10792	Class IV	Yes	Positive control
3	*E. coli* ATCC 25922	-	No	NC1
4	*S. aureus* ATCC 25923	-	No	NC1
5	*C. necator* DSM 428	Class I	No	NC2
6	*P. aeruginosa* DSM 19880	Class II	No	NC2
7	*A. vinosum* DSM 180	Class III	No	NC2
8	*H. walsbyi* DSM 16854	Class III	No	NC2

**Table 3:** The phaR-containing strains isolated from the soil of a municipal landfill in Yangon, Myanmar.

No	Strain	Closet relative species	% Similarity
1	YGMW1	*B. thuringensis*	96
2	YGMW2	*B. thuringensis*	96
3	YGMW3	*B. thuringensis*	96
4	YGMW4	*B. thuringensis*	96
5	YGMW7	*B. thuringensis*	94
6	YGMW9	*B. thuringensis*	95
7	YGMW10	*B. thuringensis*	95
