# Supplementary material for: A Novel Nucleic Lateral Flow Assay for Screening phaR-Containing Bacillus spp
Source: J Microbiol Biotechnol. 2019 Oct 25;31(1):123–9. doi: 10.4014/jmb.1907.07045 (PMC9705695; doi:10.4014/jmb.1907.07045)
Supplement: Supplementary file 1 [file jmb-31-1-123-supple.pdf]

J. Microbiol. Biotechnol.
https://doi.org/10.4014/jmb.1907.07045

Supplementary

Fig. S1. Phylogenetic relationship of the *phaR* gene of 64 *Bacillus* species. The *phaR* gene of *Kyrpidia tusciae* was assigned as an outgroup. The numbers represented on the tree are bootstrap values expressed as a percentage of 1×10^4 replications.

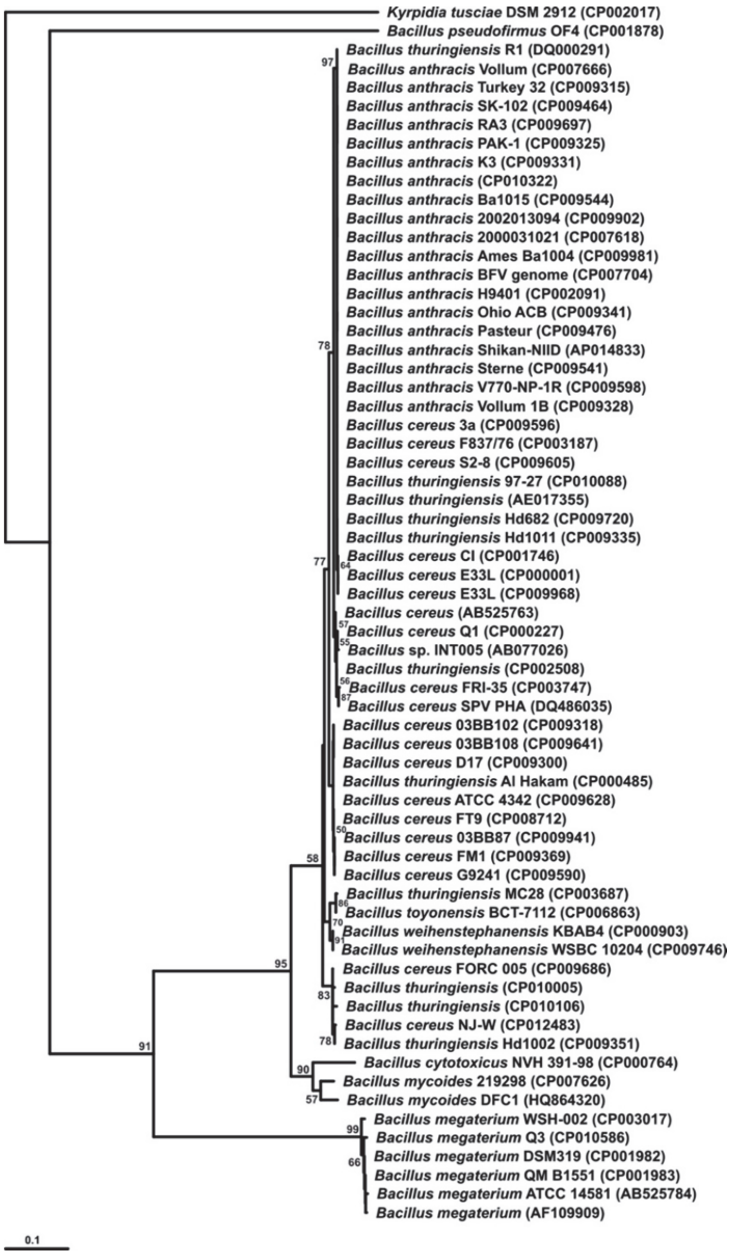

Fig. S2. Multiple sequence alignment of 63 *Bacillus* strains containing *phaR* genes. Asterisks represent conserved positions.

BGPhaR-F

```
CP007626     13 AAA---TTCGATCCACTACAAGCATGGAAAGAAGCTTATGAACAGACCGAAACATTTTGG
HQ864320     13 AAA---TTCGATCCACTACAAGCATGGAAAGAAGCTTATGAACAGACTGAAACATTTTGG
CP000764     13 AAA---TTTGATCCACTGCAAACATGGAAAGAGGTTTATGAAAAAACAGAAACGTTTTGG
CP009686     13 AAA---TTCGATCCACTACAAGCATGGAAAAATGCTTATGAACAAACCGAAACATTTTGG
CP010005     13 AAA---TTCGATCCACTACAAGCATGGAAAAATGCTTATGAACAAACCGAAACATTTTGG
CP012483     13 AAA---TTCGATCCACTACAAGCATGGAAAAATGCTTATGAACAAACCGAAACATTTTGG
CP009351     13 AAA---TTCGATCCACTACAAGCATGGAAAAATGCTTATGAACAAACCGAAACATTTTGG
CP010106     13 AAA---TTCGATCCACTACAAGCATGGAAAAATGCTTATGAACAAACCGAAACATTTTGG
CP009941     13 AAA---TTCGATCCACTACAAGCATGGAAAAATGCTTATGAACAAACCGAAACATTTTGG
CP009369     13 AAA---TTCGATCCACTACAAGCATGGAAAAATGCTTATGAACAAACCGAAACATTTTGG
CP009590     13 AAA---TTCGATCCACTACAAGCATGGAAAAATGCTTATGAACAAACCGAAACATTTTGG
CP009628     13 AAA---TTCGATCCACTACAAGCATGGAAAAATGCTTATGAACAAACCGAAACATTTTGG
CP008712     13 AAA---TTCGATCCACTACAAGCATGGAAAAATGCTTATGAACAAACCGAAACATTTTGG
CP009318     13 AAA---TTCGATCCACTACAAGCATGGAAAAATGCTTATGAACAAACCGAAACATTTTGG
CP000485     61 AAA---TTCGATCCACTACAAGCATGGAAAAATGCTTATGAACAAACCGAAACATTTTGG
CP009641     13 AAA---TTCGATCCACTACAAGCATGGAAAAATGCTTATGAACAAACCGAAACATTTTGG
CP009300     13 AAA---TTCGATCCACTACAAGCATGGAAAAATGCTTATGAACAAACCGAAACATTTTGG
CP002091     61 AAA---TTCGATCCACTACAAGCATGGAAAAATGCTTATGAACAAACCGAAACATTTTGG
AP014833     61 AAA---TTCGATCCACTACAAGCATGGAAAAATGCTTATGAACAAACCGAAACATTTTGG
CP007618     13 AAA---TTCGATCCACTACAAGCATGGAAAAATGCTTATGAACAAACCGAAACATTTTGG
CP009902     13 AAA---TTCGATCCACTACAAGCATGGAAAAATGCTTATGAACAAACCGAAACATTTTGG
CP009981     13 AAA---TTCGATCCACTACAAGCATGGAAAAATGCTTATGAACAAACCGAAACATTTTGG
CP009544     13 AAA---TTCGATCCACTACAAGCATGGAAAAATGCTTATGAACAAACCGAAACATTTTGG
CP007704     13 AAA---TTCGATCCACTACAAGCATGGAAAAATGCTTATGAACAAACCGAAACATTTTGG
CP010322     13 AAA---TTCGATCCACTACAAGCATGGAAAAATGCTTATGAACAAACCGAAACATTTTGG
CP009331     13 AAA---TTCGATCCACTACAAGCATGGAAAAATGCTTATGAACAAACCGAAACATTTTGG
CP009341     13 AAA---TTCGATCCACTACAAGCATGGAAAAATGCTTATGAACAAACCGAAACATTTTGG
CP009325     13 AAA---TTCGATCCACTACAAGCATGGAAAAATGCTTATGAACAAACCGAAACATTTTGG
CP009476     13 AAA---TTCGATCCACTACAAGCATGGAAAAATGCTTATGAACAAACCGAAACATTTTGG
CP009697     13 AAA---TTCGATCCACTACAAGCATGGAAAAATGCTTATGAACAAACCGAAACATTTTGG
CP009464     13 AAA---TTCGATCCACTACAAGCATGGAAAAATGCTTATGAACAAACCGAAACATTTTGG
CP009541     13 AAA---TTCGATCCACTACAAGCATGGAAAAATGCTTATGAACAAACCGAAACATTTTGG
CP009315     13 AAA---TTCGATCCACTACAAGCATGGAAAAATGCTTATGAACAAACCGAAACATTTTGG
CP009328     13 AAA---TTCGATCCACTACAAGCATGGAAAAATGCTTATGAACAAACCGAAACATTTTGG
CP009720     13 AAA---TTCGATCCACTACAAGCATGGAAAAATGCTTATGAACAAACCGAAACATTTTGG
CP009335     13 AAA---TTCGATCCACTACAAGCATGGAAAAATGCTTATGAACAAACCGAAACATTTTGG
CP009605     13 AAA---TTCGATCCACTACAAGCATGGAAAAATGCTTATGAACAAACCGAAACATTTTGG
CP009596     13 AAA---TTCGATCCACTACAAGCATGGAAAAATGCTTATGAACAAACCGAAACATTTTGG
CP007666     13 AAA---TTCGATCCACTACAAGCATGGAAAAATGCTTATGAACAAACCGAAACATTTTGG
CP009598     13 AAA---TTCGATCCACTACAAGCATGGAAAAATGCTTATGAACAAACCGAAACATTTTGG
CP000001     13 AAA---TTCGATCCACTACAAGCATGGAAAAATGCTTATGAACAAACCGAAACATTTTGG
CP009968     13 AAA---TTCGATCCACTACAAGCATGGAAAAATGCTTATGAACAAACCGAAACATTTTGG
CP001746     61 AAA---TTCGATCCACTACAAGCATGGAAAAATGCTTATGAACAAACCGAAACATTTTGG
CP003187     13 AAA---TTCGATCCACTACAAGCATGGAAAAATGCTTATGAACAAACCGAAACATTTTGG
DQ000291     61 AAA---TTCGATCCACTACAAGCATGGAAAAATGCTTATGAACAAACCGAAACATTTTGG
CP010088     13 AAA---TTCGATCCACTACAAGCATGGAAAAATGCTTATGAACAAACCGAAACATTTTGG
AE017355     13 AAA---TTCGATCCACTACAAGCATGGAAAAATGCTTATGAACAAACCGAAACATTTTGG
CP003747     13 AAA---TTCGATCCACTACAAGCATGGAAAAATGCTTATGAACAAACCGAAACATTTTGG
DQ486135     13 AAA---TTCGATCCACTACAAGCATGGAAAAATGCTTATGAACAAACCGAAACATTTTGG
CP002508     13 AAA---TTCGATCCACTACAAGCATGGAAAAATGCTTATGAACAAACCGAAACATTTTGG
CP000227     13 AAA---TTCGATCCACTACAAGCATGGAAAAATGCTTATGAACAAACCGAAACATTTTGG
AB077026     13 AAA---TTCGATCCACTACAAGCATGGAAAAATGCTTATGAACAAACCGAAACATTTTGG
AB525763     13 AAA---TTCGATCCACTACAAGCATGGAAAAATGCTTATGAACAAACCGAAACATTTTGG
CP003687     52 AAA---TTCGATCCATTGCAAGCATGGAAAAATGCTTATGAACAAACCGAAACATTTTGG
CP006863     13 AAA---TTCGATCCATTGCAAGCATGGAAAAATGCTTATGAACAAACCGAAACATTTTGG
CP000903     13 AAA---TTCGATCCATTGCAAGCATGGAAAAATGCTTATGAACAAACCGAAACATTTTGG
CP009746     13 AAA---TTCGATCCATTGCAAGCATGGAAAAATGCTTATGAACAAACCGAAACATTTTGG
CP001982     13 AAAGTATTTGATCCGTTTCAAGCATGGAAAGACGTATATGACAAAACCGAATCTTACTGG
AB525784     13 AAAGTATTTGATCCGTTTCAAGCATGGAAAGACGTATATGACAAAACCGAATCTTACTGG
CP001983     13 AAAGTATTTGATCCGTTTCAAGCATGGAAAGACGTATATGACAAAACCGAATCTTACTGG
AF109909     13 AAAGTATTTGATCCGTTTCAAGCATGGAAAGACGTATATGACAAAACCGAATCTTACTGG
CP010586     13 AAAGTATTTGATCCGTTTCAAGCATGGAAAGACGTATATGACAAAACCGAATCTTACTGG
CP003017     13 AAAGTATTTGATCCGTTTCAAGCATGGAAAGACGTATATGACAAAACCGAATCTTACTGG
consensus    61 ***   **.*****..*.***.********.*.*..*****..*.**.***.*.*..***
```

BGPhaR-R

```
CP007626    70 GGAAAAACGCTCAATGAAACAATCAAAACAGAAGAATATTCTGCTTGGATGGGAAGCGTT
HQ864320    70 GGAAAAGCTCTAAATGAAACAATCAAAACAGAAGAGTATTCTGCTTGGATGGGAAGCGTT
CP000764    70 GGAAAAGCATTAAATGAAACAATCAAAACAGAAGAGTATTCTGCTTGGATGGGAAGCGTT
CP009686    70 GGAAAAGCGCTCAATGAAACAATTAAAACAGAAGAATATTCTGCTTGGATGGGCAGCGTT
CP010005    70 GGAAAAGCGCTCAATGAAACAATTAAAACAGAAGAATATTCTGCTTGGATGGGCAGCGTT
CP012483    70 GGAAAAGCGCTCAATGAAACAATTAAAACAGAAGAATATTCTGCTTGGATGGGCAGCGTT
CP009351    70 GGAAAAGCGCTCAATGAAACAATTAAAACAGAAGAATATTCTGCTTGGATGGGCAGCGTT
CP010106    70 GGAAAAGCGCTCAATGAAACAATTAAAACAGAAGAATATTCTGCTTGGATGGGCAGCGTT
CP009941    70 GGAAAAGCGCTCAATGAAACAATTAAAACAGAAGAATATTCTGCTTGGATGGGCAGTGTT
CP009369    70 GGAAAAGCGCTCAATGAAACAATTAAAACAGAAGAATATTCTGCTTGGATGGGCAGTGTT
CP009590    70 GGAAAAGCGCTCAATGAAACAATTAAAACAGAAGAATATTCTGCTTGGATGGGCAGTGTT
CP009628    70 GGAAAAGCGCTCAATGAAACAATTAAAACAGAAGAATATTCTGCTTGGATGGGCAGTGTT
CP008712    70 GGAAAAGCGCTCAATGAAACAATTAAAACAGAAGAATATTCTGCTTGGATGGGCAGTGTT
CP009318    70 GGAAAAGCGCTCAATGAAACAATTAAAACAGAAGAATATTCTGCTTGGATGGGCAGTGTT
CP000485   118 GGAAAAGCGCTCAATGAAACAATTAAAACAGAAGAATATTCTGCTTGGATGGGCAGTGTT
CP009641    70 GGAAAAGCGCTCAATGAAACAATTAAAACAGAAGAATATTCTGCTTGGATGGGCAGTGTT
CP009300    70 GGAAAAGCGCTCAATGAAACAATTAAAACAGAAGAATATTCTGCTTGGATGGGCAGTGTT
CP002091   118 GGAAAAGCGCTCAATGAAACAATTAAAACAGAAGAATATTCTGCTTGGATGGGCAGCGTT
AP014833   118 GGAAAAGCGCTCAATGAAACAATTAAAACAGAAGAATATTCTGCTTGGATGGGCAGCGTT
CP007618    70 GGAAAAGCGCTCAATGAAACAATTAAAACAGAAGAATATTCTGCTTGGATGGGCAGCGTT
CP009902    70 GGAAAAGCGCTCAATGAAACAATTAAAACAGAAGAATATTCTGCTTGGATGGGCAGCGTT
CP009981    70 GGAAAAGCGCTCAATGAAACAATTAAAACAGAAGAATATTCTGCTTGGATGGGCAGCGTT
CP009544    70 GGAAAAGCGCTCAATGAAACAATTAAAACAGAAGAATATTCTGCTTGGATGGGCAGCGTT
CP007704    70 GGAAAAGCGCTCAATGAAACAATTAAAACAGAAGAATATTCTGCTTGGATGGGCAGCGTT
CP010322    70 GGAAAAGCGCTCAATGAAACAATTAAAACAGAAGAATATTCTGCTTGGATGGGCAGCGTT
CP009331    70 GGAAAAGCGCTCAATGAAACAATTAAAACAGAAGAATATTCTGCTTGGATGGGCAGCGTT
CP009341    70 GGAAAAGCGCTCAATGAAACAATTAAAACAGAAGAATATTCTGCTTGGATGGGCAGCGTT
CP009325    70 GGAAAAGCGCTCAATGAAACAATTAAAACAGAAGAATATTCTGCTTGGATGGGCAGCGTT
CP009476    70 GGAAAAGCGCTCAATGAAACAATTAAAACAGAAGAATATTCTGCTTGGATGGGCAGCGTT
CP009697    70 GGAAAAGCGCTCAATGAAACAATTAAAACAGAAGAATATTCTGCTTGGATGGGCAGCGTT
CP009464    70 GGAAAAGCGCTCAATGAAACAATTAAAACAGAAGAATATTCTGCTTGGATGGGCAGCGTT
CP009541    70 GGAAAAGCGCTCAATGAAACAATTAAAACAGAAGAATATTCTGCTTGGATGGGCAGCGTT
CP009315    70 GGAAAAGCGCTCAATGAAACAATTAAAACAGAAGAATATTCTGCTTGGATGGGCAGCGTT
CP009328    70 GGAAAAGCGCTCAATGAAACAATTAAAACAGAAGAATATTCTGCTTGGATGGGCAGCGTT
CP009720    70 GGAAAAGCGCTCAATGAAACAATTAAAACAGAAGAATATTCTGCTTGGATGGGCAGCGTT
CP009335    70 GGAAAAGCGCTCAATGAAACAATTAAAACAGAAGAATATTCTGCTTGGATGGGCAGCGTT
CP009605    70 GGAAAAGCGCTCAATGAAACAATTAAAACAGAAGAATATTCTGCTTGGATGGGCAGCGTT
CP009596    70 GGAAAAGCGCTCAATGAAACAATTAAAACAGAAGAATATTCTGCTTGGATGGGCAGCGTT
CP007666    70 GGAAAAGCGCTCAATGAAACAATTAAAACAGAAGAATATTCTGCTTGGATGGGCAGCGTT
CP009598    70 GGAAAAGCGCTCAATGAAACAATTAAAACAGAAGAATATTCTGCTTGGATGGGCAGCGTT
CP000001    70 GGAAAAGCGCTCAATGAAACAATTAAAACAGAAGAATATTCTGCTTGGATGGGCAGCGTT
CP009968    70 GGAAAAGCGCTCAATGAAACAATTAAAACAGAAGAATATTCTGCTTGGATGGGCAGCGTT
CP001746   118 GGAAAAGCGCTCAATGAAACAATTAAAACAGAAGAATATTCTGCTTGGATGGGCAGCGTT
CP003187    70 GGAAAAGCGCTCAATGAAACAATTAAAACAGAAGAATATTCTGCTTGGATGGGCAGCGTT
DQ000291   118 GGAAAAGCGCTCAATGAAACAATTAAAACAGAAGAATATTCTGCTTGGATGGGCAGCGTT
CP010088    70 GGAAAAGCGCTCAATGAAACAATTAAAACAGAAGAATATTCTGCTTGGATGGGCAGCGTT
AE017355    70 GGAAAAGCGCTCAATGAAACAATTAAAACAGAAGAATATTCTGCTTGGATGGGCAGCGTT
CP003747    70 GGAAAAGCGCTCAATGAAACAATTAAAACAGAAGAATATTCTGCTTGGATGGGCAGCGTT
DQ486135    70 GGAAAAGCGCTCAATGAAACAATTAAAACAGAAGAATATTCTGCTTGGATGGGCAGCGTT
CP002508    70 GGAAAAGCGCTCAATGAAACAATTAAAACAGAAGAATATTCTGCTTGGATGGGCAGCGTT
CP000227    70 GGAAAAGCGCTCAATGAAACAATTAAAACAGAAGAATATTCTGCTTGGATGGGCAGCGTT
AB077026    70 GGAAAAGCGCTCAATGAAACAATTAAAACAGAAGAATATTCTGCTTGGATGGGCAGCGTT
AB525763    70 GGAAAAGCGCTCAATGAAACAATTAAAACAGAAGAATATTCTGCTTGGATGGGCAGCGTT
CP003687   109 GGAAAAGCGCTCAATGAAACAATTAAAACAGAAGAATATTCTGCTTGGATGGGCAGCGTT
CP006863    70 GGAAAAGCGCTCAATGAAACAATTAAAACAGAAGAATATTCTGCTTGGATGGGCAGCGTT
CP000903    70 GGAAAAGCGCTCAATGAAACAATTAAAACAGAAGAATATTCTGCTTGGATGGGCAGCGTT
CP009746    70 GGAAAAGCGCTCAATGAAACAATTAAAACAGAAGAATATTCTGCTTGGATGGGCAGCGTT
CP001982    73 GGTAAAGTTATTGGGGACAATATGAATCGTGAAGAATTTTCCCAGCTCATGGGAAATGTG
AB525784    73 GGTAAAGTTATTGGGGACAATATGAATCGTGAAGAATTTTCCCAGCTCATGGGAAATGTG
CP001983    73 GGTAAAGTTATTGGGGACAATATGAATCGTGAAGAATTTTCCCAGCTCATGGGAAATGTG
AF109909    73 GGTAAAGTTATTGGGGACAATATGAATCGTGAAGAATTTTCCCAGCTCATGGGAAATGTG
CP010586    73 GGTAAAGTTATTGGGGACAATATGAATCGTGAAGAATTTTCCCAGCTCATGGGAAATGTG
CP003017    73 GGTAAAGTTATTGGGGACAATATGAATCGTGAAGAATTTTCCCAGCTAATGGGAAATGTG
consensus  121 **.***....*....**.*..**.**....*****.*.***.......*****.*..**.
```

BGPhaR-R

```
CP007626   130 CTAGATTTGAATTTGTTTTATCAGAAGATGTTGAATGATGCAACAAAAGGCTATTTAGAA
HQ864320   130 CTAGATTTGAACTTGTTTTATCAGAAGATGTTAAATGATGCAACAAAGGGCTATTTAGAA
CP000764   130 CTAGATTTGAACTTGTTTTATCAGAAAATGTTAAATGATGTAACGAAGGGCTATTTAGAA
CP009686   130 CTAGATTTGAATTTGTTTTATCAAAAAGCATTAAATGATACGACAAAAAACTATTTAGAA
CP010005   130 CTAGATTTGAATTTGTTTTATCAAAAAGCATTAAATGATACGACAAAAAGCTATTTAGAA
CP012483   130 CTAGATTTGAATTTGTTTTATCAAAAAGCATTAAATGATACGACAAAAAACTATTTAGAA
CP009351   130 CTAGATTTGAATTTGTTTTATCAAAAAGCATTAAATGATACGACAAAAAACTATTTAGAA
CP010106   130 CTAGATTTGAATTTGTTTTATCAAAAAGCATTAAATGATACGACAAAAAACTATTTAGAA
CP009941   130 CTAGATTTGAATTTGTTTTATCAAAAAGCATTAAATGATACAACAAAAAACTATTTAGAG
CP009369   130 CTAGATTTGAATTTGTTTTATCAAAAAGCATTAAATGATACAACAAAAAACTATTTAGAG
CP009590   130 CTAGATTTGAATTTGTTTTATCAAAAAGCATTAAATGATACAACAAAAAACTATTTAGAG
CP009628   130 CTAGATTTGAATTTGTTTTATCAAAAAGCATTAAATGATACAACAAAAAACTATTTAGAG
CP008712   130 CTAGATTTGAATTTGTTTTATCAAAAAGCATTAAATGATACAACAAAAAACTATTTAGAG
CP009318   130 CTAGATTTGAATTTGTTTTATCAAAAAGCATTAAATGATACAACAAAAAACTATTTAGAG
CP000485   178 CTAGATTTGAATTTGTTTTATCAAAAAGCATTAAATGATACAACAAAAAACTATTTAGAG
CP009641   130 CTAGATTTGAATTTGTTTTATCAAAAAGCATTAAATGATACAACAAAAAACTATTTAGAG
CP009300   130 CTAGATTTGAATTTGTTTTATCAAAAAGCATTAAATGATACAACAAAAAACTATTTAGAG
CP002091   178 CTAGATTTGAATTTGTTTTATCAAAAAGCATTAAATGATACGACAAAAAATTATTTAGAG
AP014833   178 CTAGATTTGAATTTGTTTTATCAAAAAGCATTAAATGATACGACAAAAAATTATTTAGAG
CP007618   130 CTAGATTTGAATTTGTTTTATCAAAAAGCATTAAATGATACGACAAAAAATTATTTAGAG
CP009902   130 CTAGATTTGAATTTGTTTTATCAAAAAGCATTAAATGATACGACAAAAAATTATTTAGAG
CP009981   130 CTAGATTTGAATTTGTTTTATCAAAAAGCATTAAATGATACGACAAAAAATTATTTAGAG
CP009544   130 CTAGATTTGAATTTGTTTTATCAAAAAGCATTAAATGATACGACAAAAAATTATTTAGAG
CP007704   130 CTAGATTTGAATTTGTTTTATCAAAAAGCATTAAATGATACGACAAAAAATTATTTAGAG
CP010322   130 CTAGATTTGAATTTGTTTTATCAAAAAGCATTAAATGATACGACAAAAAATTATTTAGAG
CP009331   130 CTAGATTTGAATTTGTTTTATCAAAAAGCATTAAATGATACGACAAAAAATTATTTAGAG
CP009341   130 CTAGATTTGAATTTGTTTTATCAAAAAGCATTAAATGATACGACAAAAAATTATTTAGAG
CP009325   130 CTAGATTTGAATTTGTTTTATCAAAAAGCATTAAATGATACGACAAAAAATTATTTAGAG
CP009476   130 CTAGATTTGAATTTGTTTTATCAAAAAGCATTAAATGATACGACAAAAAATTATTTAGAG
CP009697   130 CTAGATTTGAATTTGTTTTATCAAAAAGCATTAAATGATACGACAAAAAATTATTTAGAG
CP009464   130 CTAGATTTGAATTTGTTTTATCAAAAAGCATTAAATGATACGACAAAAAATTATTTAGAG
CP009541   130 CTAGATTTGAATTTGTTTTATCAAAAAGCATTAAATGATACGACAAAAAATTATTTAGAG
CP009315   130 CTAGATTTGAATTTGTTTTATCAAAAAGCATTAAATGATACGACAAAAAATTATTTAGAG
CP009328   130 CTAGATTTGAATTTGTTTTATCAAAAAGCATTAAATGATACGACAAAAAATTATTTAGAG
CP009720   130 CTAGATTTGAATTTGTTTTATCAAAAAGCATTAAATGATACGACAAAAAATTATTTAGAG
CP009335   130 CTAGATTTGAATTTGTTTTATCAAAAAGCATTAAATGATACGACAAAAAATTATTTAGAG
CP009605   130 CTAGATTTGAATTTGTTTTATCAAAAAGCATTAAATGATACGACAAAAAATTATTTAGAG
CP009596   130 CTAGATTTGAATTTGTTTTATCAAAAAGCATTAAATGATACGACAAAAAATTATTTAGAG
CP007666   130 CTAGATTTGAATTTGTTTTATCAAAAAGCATTAAATGATACGACAAAAAATTATTTAGAG
CP009598   130 CTAGATTTGAATTTGTTTTATCAAAAAGCATTAAATGATACGACAAAAAATTATTTAGAG
CP000001   130 CTAGATTTGAATTTGTTTTATCAAAAAGCATTAAATGATACGACAAAAAATTATTTAGAG
CP009968   130 CTAGATTTGAATTTGTTTTATCAAAAAGCATTAAATGATACGACAAAAAATTATTTAGAG
CP001746   178 CTAGATTTGAATTTGTTTTATCAAAAAGCATTAAATGATACGACAAAAAATTATTTAGAG
CP003187   130 CTAGATTTGAATTTGTTTTATCAAAAAGCATTAAATGATACGACAAAAAATTATTTAGAG
DQ000291   178 CTAGATTTGAATTTGTTTTATCAAAAAGCATTAAATGATACGACAAAAAATTATTTAGAG
CP010088   130 CTAGATTTGAATTTGTTTTATCAAAAAGCATTAAATGATACGACAAAAAATTATTTAGAG
AE017355   130 CTAGATTTGAATTTGTTTTATCAAAAAGCATTAAATGATACGACAAAAAATTATTTAGAG
CP003747   130 CTAGATTTGAATTTGTTTTATCAAAAAGCATTAAATGATACGACAAAAAATTATTTAGAG
DQ486135   130 CTAGATTTGAATTTGTTTTATCAAAAAGCATTAAATGATACGACAAAAAATTATTTAGAG
CP002508   130 CTAGATTTGAATTTGTTTTATCAAAAAGCATTAAATGATACGACAAAAAATTATTTAGAG
CP000227   130 CTAGATTTGAATTTGTTTTATCAAAAAGCATTAAATGATACGACAAAAAATTATTTAGAG
AB077026   130 CTAGATTTGAATTTGTTTTATCAAAAAGCATTAAATGATACGACAAAAAACTATTTAGAG
AB525763   130 CTAGATTTGAATTTGTTTTATCAAAAAGCATTAAATGATACGACAAAAAATTATTTAGAG
CP003687   169 CTAGACTTGAATTTGTTTTATCAAAAAGCATTAAATGATACGACAAAAAGTTATTTAGAA
CP006863   130 CTAGACTTGAATTTGTTTTATCAAAAAGCATTAAATGATACGACAAAAAGTTATTTAGAA
CP000903   130 CTAGATTTGAATTTGTTTTATCAAAAAGCATTAAATGATACGACAAAAAATTATTTAGAA
CP009746   130 CTAGATTTGAATTTGTTTTATCAAAAAGCATTAAATGATACGACAAAAAATTATTTAGAA
CP001982   133 CTAAATATGAACCTTCAATATCAACAAGCAGTAAATGAAGTAACGGGGCGCTATCTGCAC
AB525784   133 CTAAATATGAACCTTCAATATCAACAAGCAGTAAATGAAGTAACGGGGCGCTATCTGCAC
CP001983   133 CTAAATATGAACCTTCAATATCAACAAGCAGTAAATGAAGTAACGGGGCGCTATCTGCAC
AF109909   133 CTAAATATGAACCTTCAATATCAACAAGCAGTAAATGAAGTAACGGGGCGCTATCTGCAC
CP010586   133 CTAAATATGAACCTTCAATATCAACAAGCAGTAAATGAAGTAACGGGGCGCTATCTGCAC
CP003017   133 CTAAATATGAACCTTCAATATCAACAAGCAGTAAATGAAGTAACGGGGCGCTATCTGCAC
consensus  181 ***.*..****..*....*****..*.....*.*****....**.......***.*..*.
```

Table S1. Microorganisms for the phylogenetic analysis and primers design.

**No.**	**Organism**	**Accession No**	**Strain**	**Family**	***phaR* (bp)**
**1**	*B. anthracis*	CP 007618	2000031021	*Bacillaceae*	483
**2**	*B. anthracis*	CP 009902	2002013094	*Bacillaceae*	483
**3**	*B. anthracis*	CP 009981	Ames_BA1004	*Bacillaceae*	483
**4**	*B. anthracis*	CP 009544	BA 1015	*Bacillaceae*	483
**5**	*B. anthracis*	CP 007704	BFV genome	*Bacillaceae*	483
**6**	*B. anthracis*	CP 010322	Canadian_bison	*Bacillaceae*	483
**7**	*B. anthracis*	CP 002091	H 9401	*Bacillaceae*	531
**8**	*B. anthracis*	CP 009331	K 3	*Bacillaceae*	483
**9**	*B. anthracis*	CP 009341	Ohio ACB	*Bacillaceae*	483
**10**	*B. anthracis*	CP 009325	PAK-1	*Bacillaceae*	483
**11**	*B. anthracis*	CP 009476	Pasteur	*Bacillaceae*	483
**12**	*B. anthracis*	CP 009697	RA 3	*Bacillaceae*	483
**13**	*B. anthracis*	AP 014833	Shikan-NIID	*Bacillaceae*	531
**14**	*B. anthracis*	CP 009464	SK-102	*Bacillaceae*	483
**15**	*B. anthracis*	CP 009541	Sterne	*Bacillaceae*	483
**16**	*B. anthracis*	C P009315	Turkey 32	*Bacillaceae*	483
**17**	*B. anthracis*	CP 009598	V770-NP-1R	*Bacillaceae*	483
**18**	*B. anthracis*	C P007666	Vollum	*Bacillaceae*	483
**19**	*B. anthracis*	CP 009328	Vollum 1B	*Bacillaceae*	483
**20**	*B. cereus*	CP 009941	03BB87	*Bacillaceae*	483
**21**	*B. cereus*	CP 009318	03BB102	*Bacillaceae*	483
**22**	*B. cereus*	CP 009641	03BB108	*Bacillaceae*	483
**23**	*B. cereus*	CP 009596	3 a	*Bacillaceae*	483
**24**	*B. cereus*	CP 009628	ATCC 4342	*Bacillaceae*	483

Table S1. (cont.)

**No.**	**Organism**	**Accession No**	**Strain**	**Family**	***phaR* (bp)**
**25**	*B. cereus*	CP 001746	CI	*Bacillaceae*	531
**26**	*B. cereus*	CP 009300	D 17	*Bacillaceae*	483
**27**	*B. cereus*	CP 000001	E 33L	*Bacillaceae*	483
**28**	*B. cereus*	CP 009968	E 33L	*Bacillaceae*	483
**29**	*B. cereus*	CP 003187	F 837/76	*Bacillaceae*	483
**30**	*B. cereus*	CP 009369	FM1	*Bacillaceae*	483
**31**	*B. cereus*	CP 009686	FORC_005	*Bacillaceae*	483
**32**	*B. cereus*	CP 003747	FRI-35	*Bacillaceae*	483
**33**	*B. cereus*	CP 008712	FT 9	*Bacillaceae*	483
**34**	*B. cereus*	CP 009590	G 9241	*Bacillaceae*	483
**35**	*B. cereus*	CP 012483	NJ-W	*Bacillaceae*	483
**36**	*B. cereus*	CP 000227	Q1	*Bacillaceae*	483
**37**	*B. cereus*	CP 009605	S 2-8	*Bacillaceae*	483
**38**	*B. cereus*	DQ 486135	SPV PHA	*Bacillaceae*	483
**39**	*B. cereus*	AB 525763	-	*Bacillaceae*	483
**40**	*B. cytotoxicus*	CP 000764	NVH 391-98	*Bacillaceae*	483
**41**	*B. megaterium*	CP 001982	DSM 319	*Bacillaceae*	600
**42**	*B. megaterium*	CP 010586	Q 3	*Bacillaceae*	600
**43**	*B. megaterium*	CP 001983	QM B 1551	*Bacillaceae*	600
**44**	*B. megaterium*	AB 525784	ATCC 14581	*Bacillaceae*	600
**46**	*B. megaterium*	AF 109909	PHA gene cluster	*Bacillaceae*	600
**47**	*B. mycoides*	CP 007626	219298	*Bacillaceae*	483
**48**	*B. mycoides*	HQ 864320	DFC 1	*Bacillaceae*	483
**49**	*B. pseudofirmus*	CP 001878	OF 4	*Bacillaceae*	498
**50**	*B. thuringiensis*	CP 010088	97-27	*Bacillaceae*	483

Table S1. (cont.)

No.	Organism	Accession No	Strain	Family	*phaR* (bp)
**51**	*B. thuringiensis*	AE 017355	97-27	*Bacillaceae*	483
**52**	*B. thuringiensis*	CP 000485	Al Hakam	*Bacillaceae*	531
**53**	*B. thuringiensis*	CP 010005	HD 1	*Bacillaceae*	483
**54**	*B. thuringiensis*	CP 010106	HD 521	*Bacillaceae*	483
**55**	*B. thuringiensis*	CP 009720	HD 682	*Bacillaceae*	483
**56**	*B. thuringiensis*	CP 009351	HD 1002	*Bacillaceae*	483
**57**	*B. thuringiensis*	CP 009335	HD 1011	*Bacillaceae*	483
**58**	*B. thuringiensis*	CP 003687	MC 28	*Bacillaceae*	522
**59**	*B. thuringiensis*	CP 002508	YBT-020	*Bacillaceae*	483
**60**	*B. thuringiensis*	DQ 000291	R1	*Bacillaceae*	531
**61**	*B. toyonensis*	CP 006863	BCT-7112	*Bacillaceae*	483
**62**	*B. weihenstephanensis*	CP 000903	KBAB4	*Bacillaceae*	483
**63**	*B. weihenstephanensis*	CP 009746	WSBC 10204	*Bacillaceae*	483
**64**	*Kyrpidia tusciae*	CP 002017	DSM 2912	*Alicyclobacillaceae*	441
**65**	*Bacillus* sp.	AB 077026	INT 005	*Bacillaceae*	483
